# Lifting Machine

**Published:** 1876-01

**Authors:** 


### THE LIFTING-MACHINE.

We have commenced a regular system of daily lifting on one of Rev. Mr. Mann's "Reactionary Lifters," at the rooms of the Company, No. 46 East Fourteenth Street. A lady of our family has also entered upon the same daily duty. We have taken this step for two reasons. First, because we feel the need of more physical exercise than we are able to obtain in our sedentery city life; and secondly, because we wish to tell our readers, from time to time, just what a practical knowledge of this system teaches us. Already we discover a marked exhilaration of spirits and buoyancy of feelings, following the few minutes' daily practice. A certain elasticity of mind and body appears to be imparted by this concentration of exercise; and this alone would seem to render the system altogether desirable to such as are compelled by duty to exercise the brain largely and continuously. But we are of the opinion that one of the chief values of this system rests in its power to induce activity in the organs of digestion. There is something rather remarkable about this. That the cautious use of the Reactionary Lifter should regulate the bowels by inducing a healthy and normal action in organs long inclined to inactivity seems somewhat strange; but such is clearly the fact. We shall watch the result of this process upon ourselves with interest. We commenced by lifting 150 lbs. The lady began at 50 lbs. We are both gradually increasing; and if our daily visits are not interfered with, we hope to add some valuable testimony in our February number.

